# Bone-targeted methotrexate–alendronate conjugate inhibits osteoclastogenesis *in vitro* and prevents bone loss and inflammation of collagen-induced arthritis *in vivo*

**DOI:** 10.1080/10717544.2017.1422295

**Published:** 2018-01-05

**Authors:** Zi’ang Xie, Guanxiong Liu, Pan Tang, Xuewu Sun, Shuai Chen, An Qin, Peizhi Zhu, Jianfeng Zhang, Shunwu Fan

**Affiliations:** ^a^ Department of Orthopaedics, Sir Run Run Shaw Hospital, School of Medicine, Zhejiang University Hangzhou China; ^b^ Key Laboratory of Biotherapy of Zhejiang Province Hangzhou China; ^c^ School of Chemistry and Chemical Engineering, Yangzhou University Jiangsu China; ^d^ Department of Orthopaedics, Shanghai Key Laboratory of Orthopaedic Implant, Shanghai Ninth People’s Hospital, Shanghai Jiaotong University School of Medicine Shanghai PR China

**Keywords:** Rheumatoid arthritis, bone targeting, methotrexate, alendronate, therapeutic

## Abstract

Rheumatoid arthritis (RA), a disease that causes joint destruction and bone erosion, is related to osteoclast activity. RA is generally treated with methotrexate (MTX). In this study, a MTX–Alendronate (ALN) conjugate was synthesized and characterized. The conjugate dramatically inhibited osteoclast formation and bone resorption compared with MTX and ALN used alone or in combination. Due to the characteristics of ALN, the MTX–ALN conjugate can adhere to the exposed bone surface and enhance drug accumulation in the pathological region for targeted therapy against osteoclastogenesis. Additionally, MTX was rapidly released in the presence of lysozyme under mildly acidic conditions, similar to inflammatory tissue and osteoclast-surviving conditions, which contributes to inflammatory inhibition; this was confirmed by the presence of pro-inflammatory cytokines. Our study highlights the use of the MTX–ALN conjugate as a potential therapeutic approach for RA by targeting osteoclastogenesis.

## Introduction

Abnormal osteoclastogenesis has been reported in several metabolic bone diseases such as osteoporosis, Paget’s disease, and rheumatoid arthritis (RA) (Li et al., 2014). RA is a chronic and progressive inflammatory condition associated with bone erosion, bone loss, and joint destruction. The pathogenesis of bone erosion and systemic bone mass loss observed in patients with RA and in animal models of inflammatory arthritis, such as collagen-induced arthritis (CIA), has provided powerful evidence that osteoclasts (OCs) play an important role in structural joint damage occurring in inflammatory arthritis (Goldring & Gravallese, 2000; Redlich et al., 2002; Schett 2007; Schett & Gravallese, 2012). Synovial cells and an abundance of inflammatory cells in the affected joints secrete the receptor activator for NF-κB ligand (RANKL), which promotes OC differentiation by binding to the RANK receptor. Thus, RANKL plays an important role in OC development during arthritis progression (Schett & Gravallese, 2012; Gu et al., 2014). Furthermore, RANKL levels are positively related to the extent of destruction of the articular cartilage and subchondral bone in RA patients and collagen-induced arthritis models (Pettit et al., 2001; Stolina et al., 2005). Therefore, RA-related destruction of the bone is believed to be primarily mediated by OCs.

Given the role of OCs in RA-related bone destruction, the inhibition of OC activity has become an important therapeutic target (Redlich et al., 2002). Bisphosphonates are well-known inhibitors of OC activity and are widely used to treat osteoporosis. Among them, alendronate (ALN) and zoledronic acid (ZA) are third-generation bisphosphonates that have been shown to potently inhibit osteoclastogenesis (Wang et al., 2011; Abe et al., 2015; Fazil et al., 2015). Methotrexate (MTX), on the other hand, is conventionally used to treat RA and is effective against inflammatory symptoms as well as in the prevention or reduction of bone erosion (Strand et al., 1999). The combination of MTX and ZA has been reported to prevent bone erosion and bone loss in collagen-induced arthritis, and more strongly inhibited bone resorption than did treatment with MTX or ZA alone (Le Goff et al., 2009). However, this treatment modality has limitations because ZA reduces the anti-inflammatory effect of MTX on RA, and MTX induces severe drug resistance and its multiple adverse effects should be considered (Mount & Featherstone, 2005; Navarro & Senior, 2006; Khan et al., 2012; Chan & Rajakumar, 2014). Therefore, targeting bone erosion sites using drugs with anti-inflammatory effects and reducing nonspecific organ drug distribution may address these limitations.

Recent studies showed the advantages of nanoscale prodrugs and modified conjugates for the treatment of RA (Shin et al., 2014; Yang et al., 2017; Karacivi et al., 2017; Lee et al., 2017). In addition, bone tissues are ideal targets because of OCs, another critical factor in RA pathogenesis. Karacivi et al. (2017) demonstrated that the high mineral content of bones allows the binding of ALN conjugate through attachment of ligands with high affinity for hydroxyapatite. Based on the above evidence, in this study, we synthesized an MTX–ALN conjugate, with two conventional drugs. Our aim was to investigate its ability to inhibit OC activity and efficacy in treating inflammatory arthritis. The chemical structure, purity, and drug release behaviors of the conjugate along with its capacity to inhibit RANKL-induced osteoclastogenesis *in vitro* were investigated. Furthermore, we investigated the molecular mechanism by which the conjugate might inhibit osteoclastogenesis and evaluated its biodistribution and therapeutic efficacy in CIA mouse and rat models, respectively. Finally, radiography, pro-inflammatory cytokine analysis, and histopathology were performed to assess the anti-inflammatory properties and bone resorption efficacy of the MTX–ALN conjugate.

## Methods

### Materials

Methotrexate was obtained from Aladdin (Shanghai, CN, USA). Acetone was obtained from Sinopharm Chemical Reagent Co., Ltd (Shanghai, CN, USA). 2-Morpholinoethane-sulfonic acid (MES), *N*-hydroxysuccinimide (NHS), and 1-(3-dimethylaminopropyl)-3-ethylcarbodiimide hydrochloride (EDC) were obtained from Energy Chemical Co. (Shanghai, CN, USA). ALN was purchased from Lullaby Pharmaceutical Chemical Co. (Wuhan, Hubei, CN, USA). All solutions were prepared using ultra-pure water from a Milli-Q water purification system (Millipore Corporation, Billerica, MA, USA). Dialysis bags were obtained from UC Union Carbide (Danbury, CT, USA). The purified solution was lyophilized by LYOQUEST-55 (Azibil Telstar Technologies S.L.U., Spain). The alpha modification of Eagle’s medium (α-MEM), penicillin/streptomycin, and fetal bovine serum (FBS) were purchased from Gibco-BRL (Gaithersburg, MD, USA). A cell counting kit (CCK-8) was obtained from Dojindo Molecular Technology (Kumamoto, Japan). Recombinant mouse M-CSF and mouse RANKL were obtained from R&D (Minneapolis, MN, USA). Rap1a antibody (sc-1482) was purchased from Santa Cruz Biotechnology (Santa Cruz, CA, USA), while all other antibodies were obtained from Cell Signaling Technology (Cambridge, MA, USA). The tartrate-resistant acid phosphatase (TRAP) staining kit and all other reagents were purchased from Sigma Aldrich, unless otherwise stated.

### Preparation and characterization of MTX–ALN conjugate

Dried MTX (0.6882 g, 1.50 mmol) was dissolved in dimethylformamide (DMF 20 mL), and SOCl2 (0.357 g, 3.00 mmol) was slowly dropped into the solution. After stirring for 1 h, dried ALN (0.407 g, 1.50 mmol) was added and the mixture was stirred at 130 °C and refluxed for 2 h. The resulting solution was poured into a 300 Da MWCO dialysis bag and dialyzed in a large beaker using ultrapure water for 24 h, with the water changed every 2–6 h. Next, the material in the dialysis bag was freeze-dried to yield the target product. Cy5.5 was conjugated with MTX and MTX–ALN conjugate at a molar ratio of 1:1. Cy5.5 (11.4 mg), MTX (5 mg), and MTX–ALN (7.78 mg) were sufficiently dissolved. Then, Cy5.5 was slowly added into the other two solutions before continuous stirring for 6 h. The reaction vial was wrapped with aluminum foil, and the mixture was kept at room temperature (25 °C). Subsequently, the mixture was dialyzed in phosphate-buffered saline (PBS) solution (pH = 7.4, 10 mM; MWCO = 1000 Da). A final volume of 10.0 mL Cy5.5-labeled solution was obtained in each group.

### MTX release in vitro

The *in vitro* release of MTX from MTX–ALN conjugate was assessed in PBS at pH 7.4 or pH 4 in the presence or absence of lysozyme. MTX–ALN conjugate was dissolved in 10.0 mL PBS before transferring to a dialysis bag (MWCO = 500 Da). Next, the dialysis bag was immersed in 100 mL PBS containing 5% (v/v) Tween-80 at 37 °C with continuous stirring at 100 rpm. At predetermined time intervals, 2.0 mL of release medium was collected for testing, and an equal volume of rat plasma containing 5% (v/v) Tween-80 was added to the system. The amount of MTX released was determined by UV–Vis spectrophotometer (UV-1800, Shimadzu, Kyoto, Japan) at *λ*_abs_ = 302 nm. The experiments were carried out in triplicate.

### Hydroxyapatite (HA) binding assay

Methotrexate and methotrexate –alendronate conjugate containing equal amounts of MTX (0.5 mg) were dissolved in PBS. The conjugate solution was incubated with HA powder (200 mg) at 37 °C as described previously (Karacivi et al., 2017). At predetermined time points, samples were centrifuged at 6000*g* for 2 min and supernatants were collected. Absorbance at 302 nm were measured to detect unbound MTX.

The HA binding capacity was estimated using the following formula:

$$\%\;Binding=\frac{A_{(MTX)0}{-A}_{(MTX)t}}{A_{(MTX)0}}\times 100$$

### Mouse bone marrow macrophage (BMM) preparation and OC differentiation

Primary bone marrow macrophages (BMMs) were isolated from the whole bone marrow of male, six-week-old C57BL/6 mice. Cells were isolated from the femoral and tibial bone marrow and cultured in α-MEM supplemented with 10% FBS, 1% penicillin/streptomycin, and 30 ng/mL M-CSF in a 37 °C, 5% CO_2_ incubator until reaching 90% confluence. The BMMs were seeded into a 96-well plate at a density of 8 × 10^3^ cells/well, in triplicate, in the presence of 30 ng/mL M-CSF, 50 ng/mL RANKL, and different concentrations of MTX (0.125, 1, 4 μM), ALN (0.125, 1, 4 μM), MTX and ALN (0.125, 1, 4 μM), or MTX–ALN conjugate (0.125, 1, 4 μM). Cells were cultured for seven days, washed twice with PBS, fixed with 4% paraformaldehyde for 20 min, and stained for TRAP. TRAP-positive cells with more than three nuclei were counted under a microscope.

### Cell viability assay

The cytotoxic effects of MTX–ALN, MTX, and ALN on BMMs were determined using a CCK-8 assay. BMMs were plated in 96-well plates at a density of 2 × 10^4^ cells/well, in triplicate, in the presence of 30 ng/mL M-CSF for 24 h. Cells were then separately treated with different concentrations of MTX–ALN (0, 0.125, 0.25, 0.5, 1.25, 2.5, 5, 10, 20, and 40 μM), MTX (0, 0.125, 0.25, 0.5, 1.25, 2.5, 5, and 10 μM), or ALN (0, 0.5, 1.25, 2.5, 5, 10, 20, and 40 μM) for 48 or 96 h. Next, 10 μL of CCK-8 buffer was added to each well, and plates were incubated for an additional 1 h. Absorbance was measured at a wavelength of 450 nm (650 nm reference) on an ELX800 absorbance microplate reader (Bio-Tek Instr., Winooski, VT, USA).

### Resorption pit assay

Bone marrow macrophages were seeded at a density of 8 × 10^3^ cells/well onto bovine bone slices in a 96-well plate in triplicate. After 24 h, cells were treated with 50 ng/mL RANKL, 30 ng/mL M-CSF, and 0, 0.125, 1.25, or 2 μM MTX–ALN, while other wells were treated with 0, 0.125, 1.25, or 2 μM MTX or 0, 0.125, 1.25, or 2 μM ALN, or both MTX and ALN until mature OCs formed. Cells were then fixed with 2.5% glutaraldehyde. Resorption pits were visualized under a scanning electron microscope (FEI Instr., Hillsboro, OR, USA), and the bone resorption area was quantified using Image J software (National Institutes of Health, Bethesda, MD, USA).

### RNA extraction and quantitative RT-PCR assay

Total RNA was extracted using an RNeasy Mini Kit (Qiagen, Valencia, CA, USA). Complementary DNA (cDNA) was synthesized using 1 μg of RNA from each sample, 2 μL of 5 × PrimeScript RT Master Mix (Takara Bio, Otsu, Japan), and 4 μL of RNase-free dH_2_O in a total volume of 10 μL. Real-time reverse transcriptase polymerase chain reaction (RT-PCR) was performed using an ABI Prism 7500 system (Applied Biosystems, Foster City, CA, USA) and SYBR^®^ Green QPCR Master Mix (Takara Bio, Otsu, Japan). The total volume (10 μL) of each PCR mix comprised 5 μL SYBR^®^ Green QPCR Master Mix, 3 μL ddH_2_O, 1 μL cDNA, and 10 μM each of forward and reverse primers. RT-PCR was performed at 95 °C for 10 min (activation), followed by 40 cycles of 95 °C for 10 s, 60 °C for 20 s, 72 °C for 20 s (amplification), and a final extension at 72 °C for 90 s. The quantity of each target was normalized to GAPDH. The rat primer sequences are shown in Table S1.

### In vivo biodistribution of conjugates

All animals were handled according to the protocol approved by the Institutional Animal Care and Use Committee of Sir Run Run Shaw Hospital. DBA/1 J mice were maintained in 12 h light/12 h dark cycles with continuous access to food and water. CIA was induced in male DBA/1 J mice at 8–10 weeks of age. To induce CIA, mice were intradermally injected with Collagen II (2.0 mg/mL) emulsified in equal volume of incomplete Freund’s adjuvant (IFA) (2.0 mg/mL) at the end of tail. It was followed by a booster immunization at 14 days after the primary immunization with Collagen II equally emulsified in IFA. At 48 h after primary immunization, the Cy5.5-labeled MTX and MTX–ALN conjugate were intravenously injected with a MTX equivalent dose of 5.0 mg per kg body weight (mg/(kg BW)), and free Cy5.5 dissolved in PBS was injected as a control. The mice were killed at 4 h post-injection. The inflamed joints, livers, and kidneys were excised immediately and subsequently washed with NS three times for ex vivo imaging of Cy5.5 fluorescence on a *in vivo* imaging system (IVIS Lumina LT; Perkin Elmer, Santa Clara, CA, USA). The signals were quantitatively analyzed using Living Image^®^ software (Perkin Elmer, Santa Clara, CA, USA).

### In vivo therapeutic efficacy in CIA rats

We used 25 eight-week-old female Wistar rats previously studied in this experiment, and randomly selected 20 rats as arthritis models; the others were used as the negative group (NG) (Song et al., 2015). Lyophilized native bovine type II collagen Sigma (Shanghai, China) was dissolved at a concentration of 2 mg/mL in 0.1 M acetic acid. The solution was incubated overnight at 4 °C, and 0.3 mL in a 1:1 emulsion with incomplete Freund’s adjuvant was injected intradermally into the base of the tail on days 0 and 14. Arthritis developed 15 days after the first injection. The arthritic rats were divided into four groups: injection with MTX–ALN conjugate (1 mg/kg/week), injection with MTX (1 mg/kg/week), injection with ALN (0.5 mg/kg, twice a week), and injection with MTX (1 mg/kg/week) along with ALN (0.5 mg/kg, twice a week). The positive control group was injected with PBS. Treatments were started at the onset of arthritis, which was 15 days after the first injection.

### Assessment of arthritis score and X-ray examination

The clinical score was assessed by a clinician with experience in blinding methods. The rats were examined every two days for signs of joint inflammation, and scored as follows: 0 = normal; 1 = mild swelling, and erythema confined to the midfoot and ankle joint; 2 = mild swelling, and erythema extending to the midfoot and ankle joint; 3 = moderate swelling, and erythema extending from the metatarsal joints to the ankle; or 4 = severe swelling, and erythema encompassing the foot, ankle, and digits. Paw scores were summed for each rat, giving a maximum possible score of 16 per rat (Le Goff et al., 2009). Hind footpad width was measured with calipers at baseline and twice weekly. At the end of the experiment, rats were euthanized via carbon dioxide asphyxiation and the hind paws were radiographed by X-ray.

### Micro-CT scanning

Fixed tibia and ankle joints were analyzed using a high-resolution micro-CT scanner (Skyscan 1072; Skyscan, Aartselaar, Belgium). The scanning protocol was set at an isometric resolution of 9 mm and X-ray energy settings of 80 kV and 80 mA. Bone volume/tissue volume (BV/TV), bone surface/bone volume (BS/BV), trabecular thickness (Tb.Th), trabecular number (Tb.N), trabecular separation (Tb.Sp), and porosity were measured using the resident reconstruction program (Skyscan, Aartselaar, Belgium).

### Histological analysis

Fixed ankle joints were decalcified in 10% EDTA for three weeks and then embedded in paraffin. Histological sections were prepared for hematoxylin and eosin (H&E) staining, TRAP staining, and immunocytochemistry for cathepsin K (CTSK) in OCs. The histopathological score of joints was assessed as described previously on sections stained with H&E using the following scale, 0 = normal synovium, 1 = synovial membrane hypertrophy and cell infiltrates, 2 = pannus and cartilage erosion, 3 = major erosion of cartilage and subchondral bone, and 4 = loss of joint integrity and ankylosis (Nishikawa et al., 2003). TRAP staining was performed to determine OC numbers in joint sections, and the expression of CTSK as measured by immunofluorescence was examined in each sample using Image-Pro Plus software (Media Cybernetics, Bethesda, MD, USA).

### Statistical analysis

Data are expressed as mean ± standard error of the mean (SEM). Experiments were conducted at least three times. Graphing and statistical analyzes were performed using Prism software (v6; GraphPad Software, San Diego, CA, USA). Student’s *t*-test was used to make comparisons between two groups. *p* < .05 indicated a significant difference between groups.

## Results and discussion

### Synthesis and characterization of the conjugate

**Figure 1.**
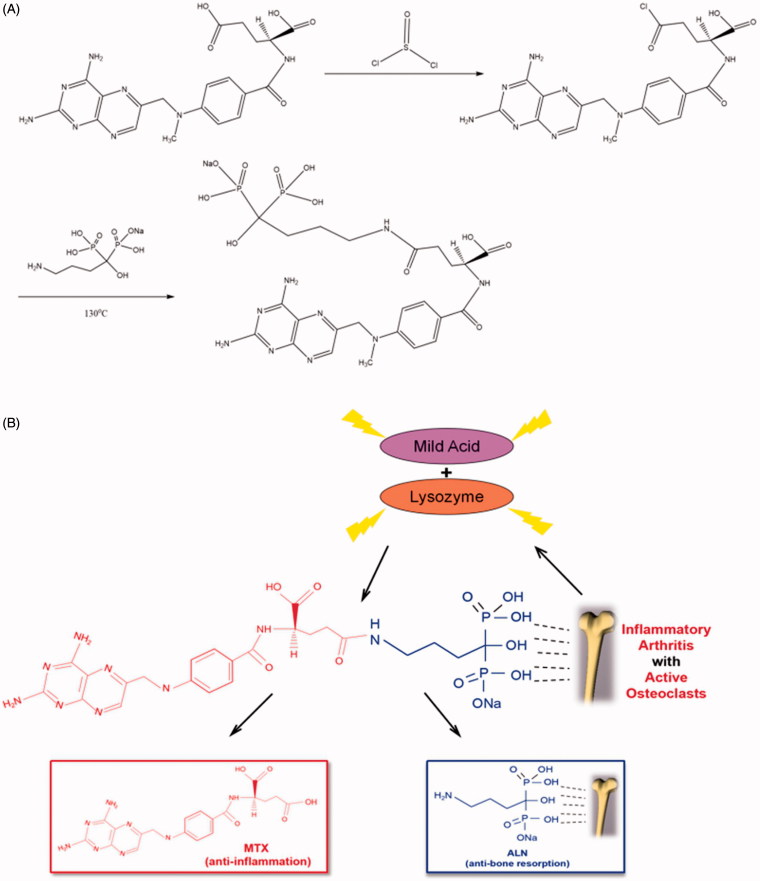
(A) Synthesis and chemical structure of conjugate. (B) Schematic illustration of MTX–ALN conjugate for selective accumulation and drug release in the presence of lysozyme and mildly acidic conditions.

As shown in Figure 1, we retained the P–C–P structure of ALN in the conjugate, which is necessary for ALN to bind to the bone tissue. Furthermore, the FT-IR spectra of MTX before and after the incorporation of ALN (Figure S1) showed that the MTX–ALN conjugate spectrum had absorption bands located at 1569 and 1724 cm^−1^, respectively, which were attributed to amide-NH bending, demonstrating that ALN had been chemically incorporated into MTX. As shown in Figure S2, the absorption of MTX was 302 nm and ALN had no UV absorption peak. Moreover, the MTX–ALN conjugate had an absorption of 302 nm, indicating that ALN had been chemically incorporated into MTX. As determined by proton nuclear magnetic resonance (^1 ^H NMR), the peak position at 4.97 ppm shown in Figure S3 is the hydroxyl group from ALN, and 7–8.8 ppm is the peak position of the aromatic ring of MTX. As shown in Figure S4, we next confirmed the ^13^C NMR spectra of MTX, ALN, and MTX–ALN. ^31 ^P NMR spectra of ALN and MTX–ALN were also obtained to confirm that ALN was indeed incorporated into MTX (Figure S5). To ensure the purity of the conjugate, we conducted LC–MS analysis of the product. As shown in Figure S6, peak 1 represents the solvent, peak 2 is the unreacted raw material MTX, peak 3 represents the reaction product, and peak 4 represents multimers of the starting material. The target molecule can be identified based on its deprotonated molecule [M-2H]^2−^ at *m/z* 705 and [M·2H_2_O + Na-2H]^−^ at *m/z* 764.

### *MTX release profiles of MTX–ALN conjugate* in vitro

**Figure 2.**
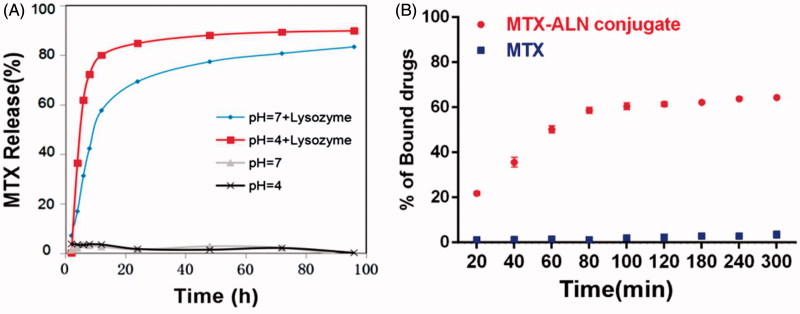
Characteristics of MTX–ALN. (A) MTX release behavior of MTX–ALN at 37 °C. (B) Binding kinetics of MTX and MTX–ALN to bone mineral HA in PBS.

Methotrexate release was evaluated out under acidic conditions or in the presence of lysozyme. As shown in Figure 2(A), MTX–ALN conjugate was incubated at 37 °C in PBS at pH 4 or 7.4 in the presence or absence of lysozyme. The level of MTX release at pH 4 and in the presence of lysozyme was slightly higher than at pH 7.4 and in the presence of lysozyme. In the absence of lysozyme, MTX was not released from the conjugate, which may have been due to the stable amide-based linkers.

### HA binding assay

To investigate the binding capacity of the conjugate to the bone tissue, its binding to HA was evaluated. As shown in Figure 2(B), 63% of the conjugate was bound to HA whereas only 3% MTX was bound to HA. The extent of binding was slightly lower than that observed in a previous study (Karacivi et al., 2017). The binding constant values of the conjugate and MTX were calculated. The correlation coefficient (*R* ^2^) of the MTX–ALN binding capacity was 0.988, but that of MTX was 0.965.

### MTX–ALN conjugate suppressed RANKL-induced OC differentiation in vitro

**Figure 3.**
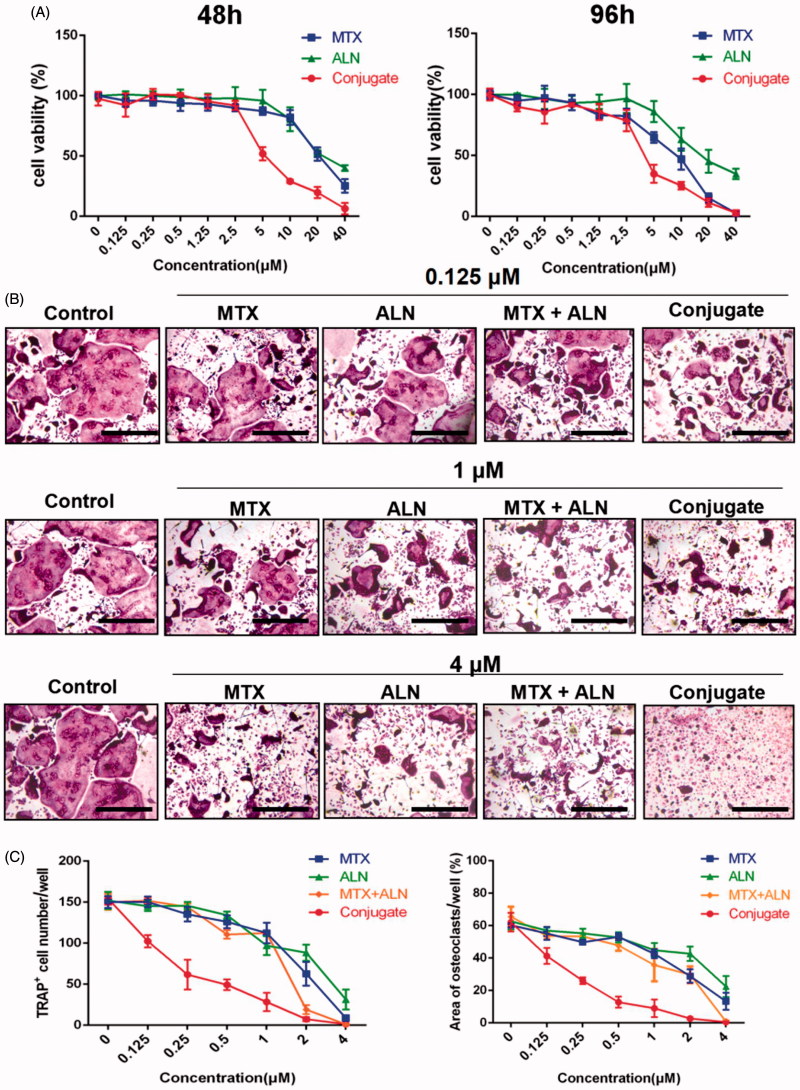
MTX, ALN, MTX with ALN, and MTX–ALN inhibited RANKL-induced osteoclastogenesis *in vitro*. (A) Viability of BMMs treated with MTX, ALN, MTX with ALN, and MTX–ALN were evaluated via CCK8 assay at 48 and 96 h. (B) BMMs were treated with the indicated concentrations of MTX, ALN, MTX with ALN, or MTX–ALN in the presence of M-CSF (30 ng/mL) and RANKL (50 ng/mL) for seven days. Cells were then fixed with 4% paraformaldehyde and stained for TRAP. (C) Number and areas of TRAP-positive multinuclear cells. Scale bar = 100 μm. MTX + ALN, free MTX combined with free ALN.

Bone erosion and bone loss are central features of RA and are associated with joint destruction and severe disability. Previous studies confirmed that OCs are the key mediators of bone destruction in RA (Okamoto & Takayanagi, 2011; Schett & Gravallese, 2012). Thus, to prevent RA progression, an ideal therapeutic drug should efficiently inhibit OC activity. Bisphosphonate directly inhibits RANKL-stimulated OC differentiation and fusion in RAW264.7 cells and has a direct effect on mature OCs by inducing their apoptosis and inhibiting their activity (Heymann et al., 2004; Wang et al., 2011; Fazil et al., 2015). MTX is used for treating RA and is effective against inflammatory symptoms as well as in the prevention or reduction of bone erosion (Cronstein, 2005; Finckh et al., 2006; Le Goff et al., 2009). Thus, we investigated the effects of the MTX–ALN conjugate on osteoclastogenesis in comparison to MTX, ALN, and MTX together with ALN. We first evaluated the effect of MTX, ALN, and MTX–ALN on OC precursor cells. Initially, we tested primary BMM viability for 48 and 96 h to determine dose toxicity. No cytotoxic effects of MTX, ALN, or MTX–ALN were observed at doses below 2.5, 5, or 0.5 μM, respectively. The IC50 values for the conjugate were 4.346 μM (48 h) and 3.546 μM (96 h) (Figure 3(A)). To determine the effect of MTX–ALN on RANKL-induced OC differentiation, we treated BMMs with RANKL and M-CSF in the presence of MTX (0.125, 1, or 4 μM), ALN (0.125, 1, or 4 μM), MTX with ALN (0.125, 1, or 4 μM each), or MTX–ALN (0.125, 1, or 4 μM). As shown in Figure 3(B), MTX–ALN significantly inhibited the formation of TRAP-positive multinucleated OCs at the lower concentration of 0.125 μM; however, MTX and ALN did not inhibit the formation of OCs at the concentration of 0.125 μM. Furthermore, MTX–ALN decreased the number of TRAP-positive cells from 156/well (control) to 20/well, and reduced the total OC area from 62.15% (control) to 12.8% at a concentration of 0.5 μM. However, 2.5 μM MTX was required to decrease the TRAP-positive cell number from 156/well (control) to 35.6/well, and to reduce the total OC area from 62.15% (control) to 18.26%. For ALN, a concentration of 4 μM was required to reduce the number of TRAP-positive cells from 156/well (control) to 26/well, and to decrease the total OC area from 62.15% (control) to 22.44% (Figure 3(C)). These data showed that MTX–ALN more effectively inhibited osteoclastogenesis than MTX, ALN, or MTX together with ALN.

### *MTX–ALN conjugate inhibited OC bone resorption* in vitro

To further investigate the role of MTX–ALN conjugate in bone resorption compared with MTX, ALN, and MTX together with ALN, BMMs plated on bovine bone slices were treated with MTX (0.125, 1.25, or 2 μM), ALN (0.125, 1.25, or 2 μM), MTX with ALN (0.125, 1.25, or 2 μM), or MTX–ALN conjugate (0.125, 1.25, or 2 μM). Reduced areas of bone resorption are shown in Figure S7(A). Areas of bone resorption for the MTX–ALN conjugate-treated group were less than those for the control group (0 μM) at a concentration of 0.125 μM. Compared to MTX with ALN group, the conjugate-treated group showed better inhibition of bone resorption. At concentrations up to 2 μM, no differences were observed on inhibition on bone resorption. Thus, MTX–ALN conjugate treatment was associated with the least bone resorption areas (Figure S7(B–D)). Taken together, these data showed that MTX–ALN conjugate more strongly inhibited bone resorption compared with the MTX with ALN group.

### *Selective biodistribution* in vivo

**Figure 4.**
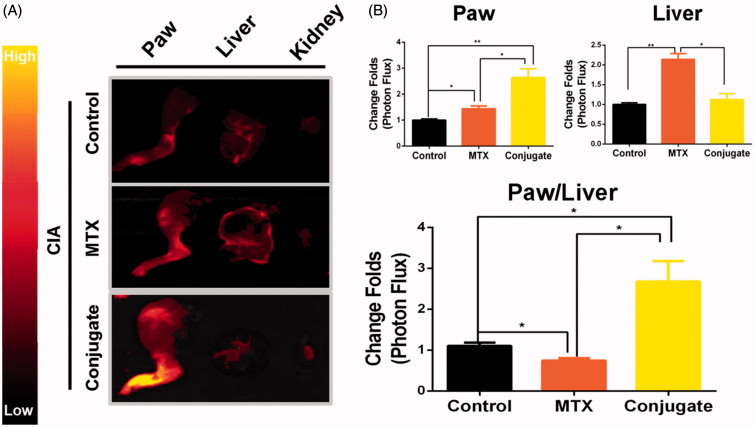
Selective biodistribution *ex vivo*. (A) Cy5.5 fluorescence images of isolated paws, livers, and kidneys. (B) Average signals detected in ankle joints of negative control mice treated with Cy5.5, CIA mice treated with Cy5.5, CIA mice treated with MTX, and CIA mice treated with conjugate. *n* = 3, *p < .05, **p < .01, ***p < .005.

Given the adverse effects of MTX and its therapeutic efficacy, its biodistribution is critical (Allen & Cullis, 2004). In the present study, CIA mice were intravenously injected with MTX or MTX–ALN conjugate 16 days after the initial injection of collagen II, with free Cy5.5 as the control. At 4 h post-injection of drugs, the hind limbs, livers, and kidneys were isolated. As shown in Figure 4(A), fluorescence imaging of paw and liver tissue sections revealed a higher accumulation of conjugate within the inflamed joints than in the liver. Quantitative analysis of fluorescence intensity in the paws and liver showed a significant increase of paw-to-liver fluorescence ratio in conjugate-injected mice compared with MTX-injected mice (Figure 4(B)). Taken together, these findings indicate that the conjugate had improved the biodistribution of MTX and showed organ-targeting potential.

### MTX–ALN conjugate attenuated RA symptoms in the CIA model

**Figure 5.**
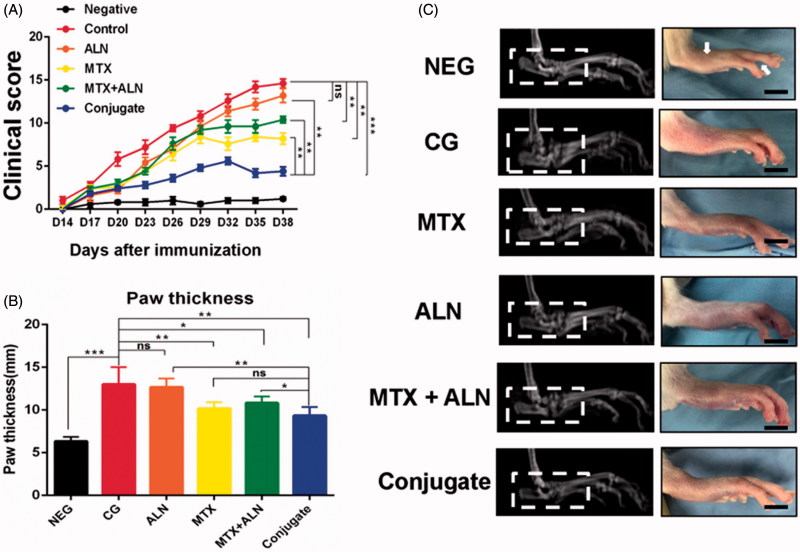
Efficiency of MTX–ALN in the CIA model. (A) Clinical scores of CIA model are shown. (B) Serial measurements of hind-paw thickness in CIA rats in the control (PBS 1 mg/kg; *n* = 5), MTX–ALN (1 mg/kg; *n* = 5), MTX (1 mg/kg; *n* = 5), ALN (100 µg/kg; *n* = 5), and MTX (1 mg/kg) with ALN (100 µg/kg) (*n* = 5) groups. (*p < .05, **p < .01, ***p < .005 compared with controls. ###*p* < .001, compared with NEG). (C) Macroscopic images of inflamed joints of NEG, CG, MTX, ALN, MTX with ALN, and MTX–ALN groups are shown. NEG: negative control; CG: control; MTX + ALN: free MTX combined with free ALN.

To explore the therapeutic efficacies of MTX, ALN, and MTX–ALN conjugate in RA, the clinical scores of treatment groups were calculated by a clinician as described above. MTX–ALN conjugate dramatically attenuated the symptoms of CIA rats compared to MTX, ALN, and MTX with ALN (Figure 5(A)). However, only the hind paw thickness in the MTX group (mean 9.5 mm) and the MTX–ALN group (mean 10.6 mm) was significantly attenuated compared with that in the control group (mean 13 mm). The ALN group (mean 12.7 mm) was not significantly different to control (Figure 5(B)). Thus, MTX and MTX–ALN treatment attenuated the symptoms of inflammation. Furthermore, radiographic examination of the hind paws of CIA rats revealed severe bone matrix resorption and erosion that suggested active arthritis and bone destruction. Bone resorption and erosion in the control group was markedly more extensive than that in the MTX group, ALN group, MTX with ALN group, or MTX–ALN conjugate group (Figure 5(C)).

### Expression of pro-inflammatory cytokines and OC-related genes

To further elucidate the anti-inflammatory and anti-bone resorption effects of MTX–ALN conjugate, RT-PCR was performed. Pro-inflammatory cytokines, such as TNFα and IL-6, are crucial indicators for the process of RA (Choy & Panayi, 2001). TNFα is involved in osteoclastogenesis and the destruction of the cartilage and bone (Feldmann & Maini, 2008). IL-6 is involved in osteoclastogenesis, the activation of osteoclasts, and bone erosion (Kim et al., 2015). In addition, NFATc1 is critical for promoting osteoclastogenesis, which promotes the transcriptional activity of TRAP (Takayanagi et al., 2000; Ishii et al., 2009). However, CTSK and DC-STAMP are involved in bone resorption to degrade the bone matrix (Takayanagi et al., 2000; Asagiri et al., 2008). As shown in Figure S8, the expressions of TNFα, IL-6, CTSK, TRAP, NFATc1, and DC-STAMP were all downregulated by MTX, ALN, MTX with ALN, and the conjugate. Compared with the other treatment groups, however, 1–10-fold downregulation of gene expression was observed in the conjugate-treated group. This showed that the conjugate had both anti-inflammatory and anti-bone resorption properties as well as selective biodistribution and targetability to the bone to release MTX.

### Bone morphometry assay

**Figure 6.**
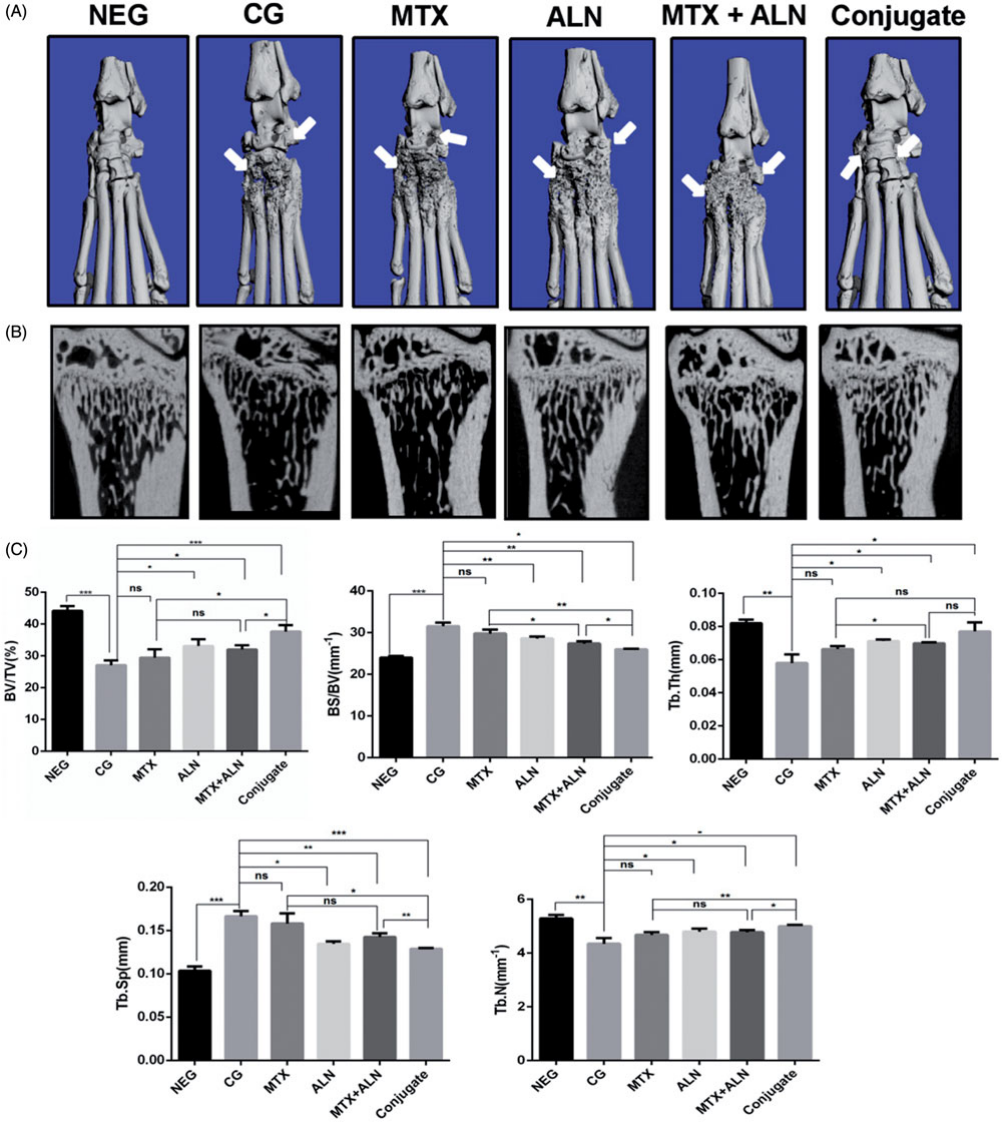
MTX–ALN prevented bone erosion and systemic bone loss in the CIA model. (A) Hind paws of rats were scanned with a high-resolution micro-CT. The most severe bone erosion was observed in the control group, while the least severe was shown in the MTX–ALN group. (B) Representative high-resolution micro-CT images of the distal tibia for each rat group. (C) Histomorphometric analysis of the distal tibia for the various treatment groups showing a significant increase in bone volume/tissue volume (BV/TV), bone surface/bone volume (BS/BV), trabecular thickness (Tb.Th.), trabecular separation (Tb.Sp.), and trabecular number (Tb.N.) in rats treated with MTX–ALN and ALN; MTX had no effect on histomorphometric parameters. ns, not significant. *p < .05, **p < .01, ***p < .005. NEG: negative control; CG: control; MTX + ALN: free MTX combined with free ALN.

The effects of all treatments on bone erosion in the periarticular bone and systemic bone loss were analyzed via micro-CT evaluation of the periarticular bone in ankle joints and the distal end of left tibiae with a quantitative histomorphometric imaging method (Figure 6(A,B)). BV/TV, BS/BV, Tb.Th., Tb.N., and Tb.Sp. were measured from the three-dimensional (3D) reconstructed images. Compared with the control group, the MTX group, ALN group, and MTX with ALN group showed significantly decreased values for BV/TV, Tb.N., and Tb.Th., and increased values of Tb.Sp. and BS/BV in the distal left tibia (Figure 6(C)). However, the MTX–ALN conjugate group showed a better prevention of bone loss than the other treatment groups did.

### Histopathology

Histological analyzes were carried out to confirm the effects of treatment on joint destruction and subchondral bones by H&E staining. Compared with the control group (mean score = 3.8), the MTX group (mean score = 2.6), ALN group (mean score = 2.8), MTX with ALN group (mean score = 2), and the conjugate group (mean score = 1) showed lower scores for ankle joints (Figure S9(A,B)). As expected, the control group showed the highest score and lower scores were observed in the treatment groups. In addition, conjugate group showed a more remarkable effect in treating CIA. The joint space became increasingly narrow, and the articular cartilage, eroded by inflammatory cytokines, was also tough. Thus, cytokine IL-6 expression of joint sections was detected by immunocytochemistry to confirm the mRNA expression of IL-6 in joints. As expected, a significant increase in the expression of IL-6 was observed in the joints of the control group, whereas CIA rats administered the conjugate showed remarkably reduced IL-6 expression. Some extent of inhibition was also observed in MTX, ALN, and MTX with ALN group. Because the mRNA expression of OC-related genes were dramatically inhibited by the conjugate, we further assess the number of OCs. Joint sections were stained with TRAP and the expression of CTSK was measured by immunocytochemistry. Semi-quantitative analysis of the MTX, ALN, MTX with ALN, and conjugate groups confirmed that the conjugate significantly reduced OC numbers compared with the MTX, ALN, and MTX with ALN groups (Figure S9(C,D)).

## Conclusions

Methotrexate and alendronate are classically used drugs for treating RA and osteoporosis, respectively. However, MTX shows adverse effects and drug resistance, while ALN possesses a desirable property of bone targeting. Thereby, in this study, we synthesized a novel bone-targeting conjugate, MTX–ALN and evaluated it for enhancement of OC inhibition. As a bone-targeting molecule, ALN was conjugated to MTX via a condensation reaction. ^1^H NMR, ^13^C NMR, ^31^P NMR, and LC–MS were used to characterize the conjugate. A remarkably preferential affinity towards the bone mineral HA was observed for the conjugate, when compared with MTX alone, and effective release of MTX in the presence of lysozyme at pH 4 was observed from the conjugate. Furthermore, remarkably improved inhibition of osteoclastogenesis and bone resorption was shown by the conjugate compared with MTX, ALN, or MTX with ALN. In summary, MTX–ALN shows significant potential as a bone-targeting conjugate for the treatment of RA. Further studies will be needed to investigate the conjugate for the treatment of metastatic bone cancer and osteoporosis. Increasing numbers of bone-targeting conjugates are expected to be developed and tested for their therapeutic efficacies in the future.

## Supplementary Material

IDRD_Fan_et_al_Supplemental_Content.pdf
