# Supplementary material for: Bone-targeted methotrexate–alendronate conjugate inhibits osteoclastogenesis in vitro and prevents bone loss and inflammation of collagen-induced arthritis in vivo
Source: Drug Deliv. 2018 Jan 5;25(1):187–97. doi: 10.1080/10717544.2017.1422295 (PMC6058523; doi:10.1080/10717544.2017.1422295)
Supplement: IDRD_Fan_et_al_Supplemental_Content.pdf [file IDRD_A_1422295_SM9982.pdf]

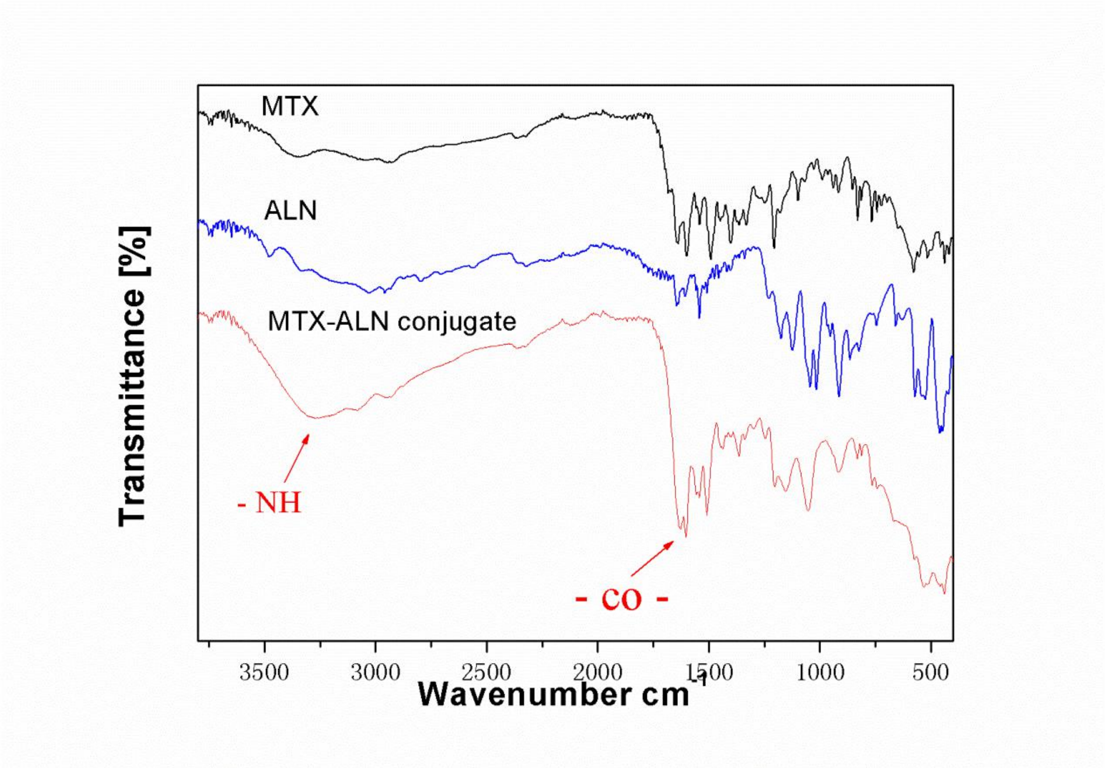

Figure S1. FT-IR spectra of MTX (black), ALN (blue), and MTX-ALN (red)

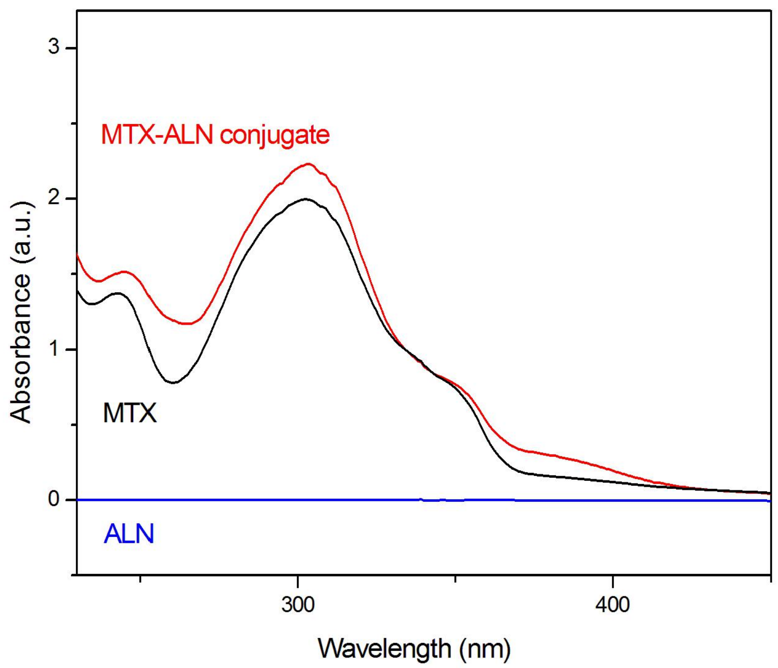

Figure S2. UV-Vis spectra of MTX, ALN, and MTX-ALN conjugate

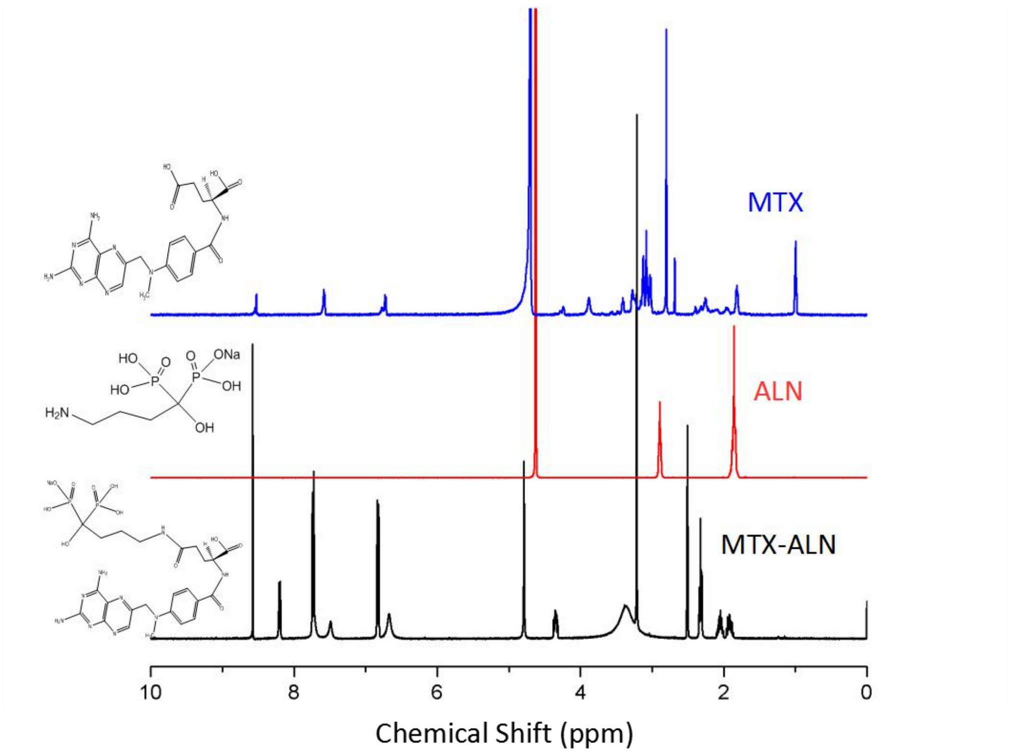

Figure S3. ^{1}H **NMR spectra of MTX (blue), ALN (red), and MTX-ALN conjugate (black)**

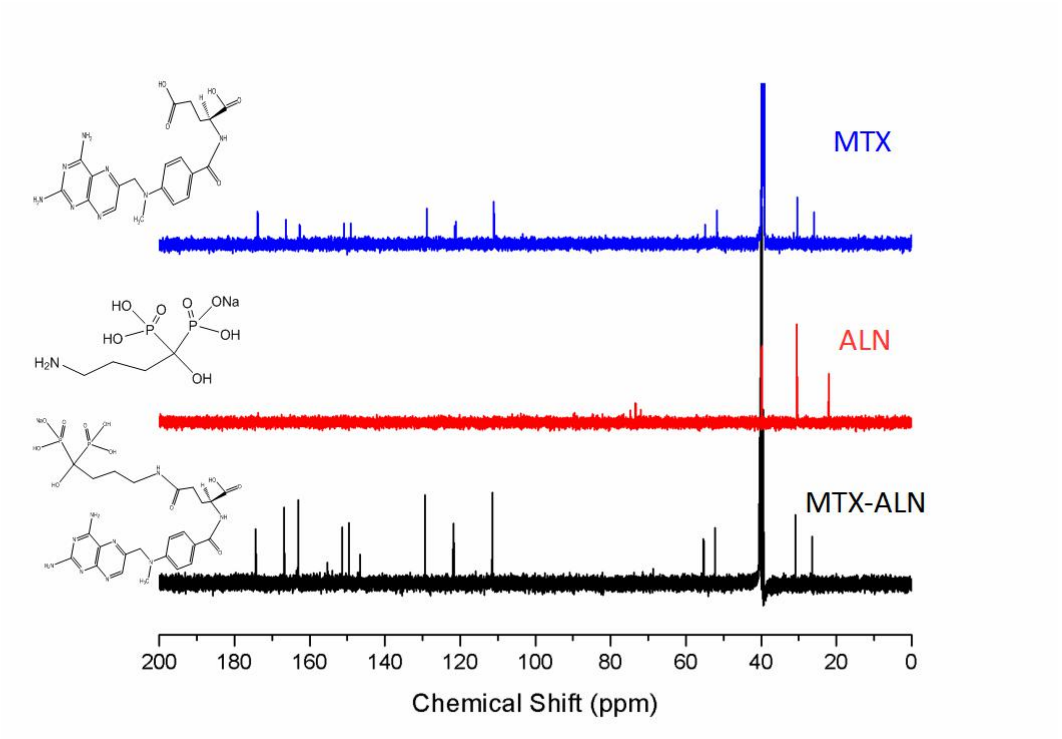

Figure S4. ^{13}C **NMR spectra of MTX (blue), ALN (red) and MTX-ALN conjugate (black)**

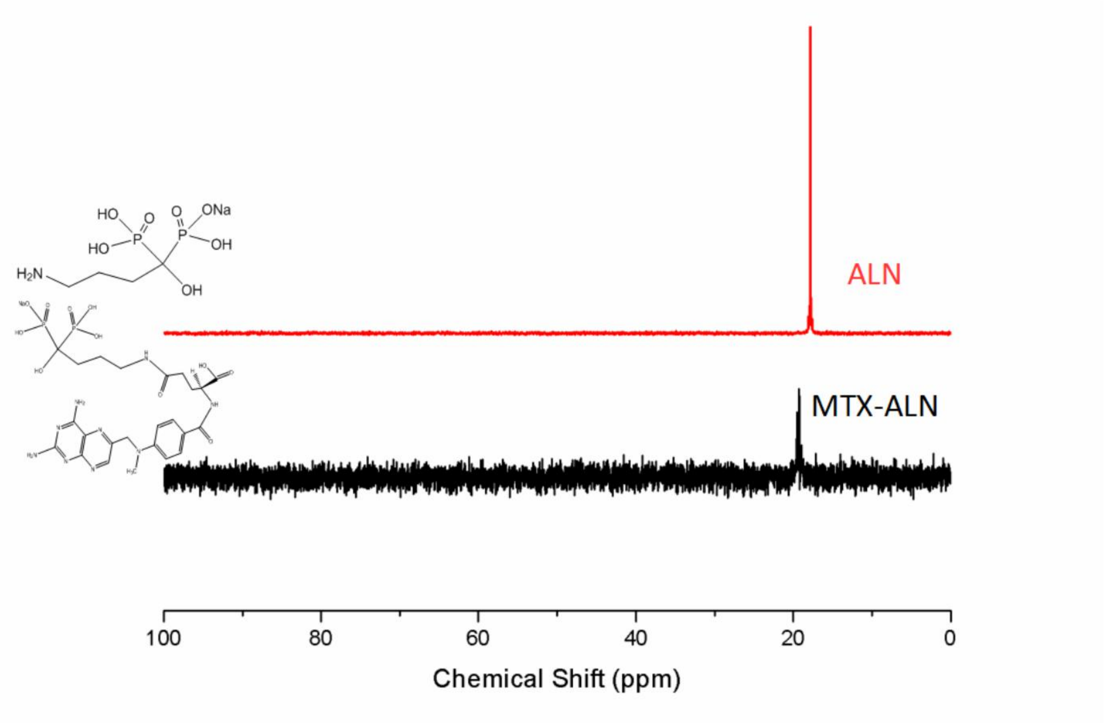

Figure S5. ^{31}P NMR spectra of ALN (red) and MTX-ALN (black) conjugate.

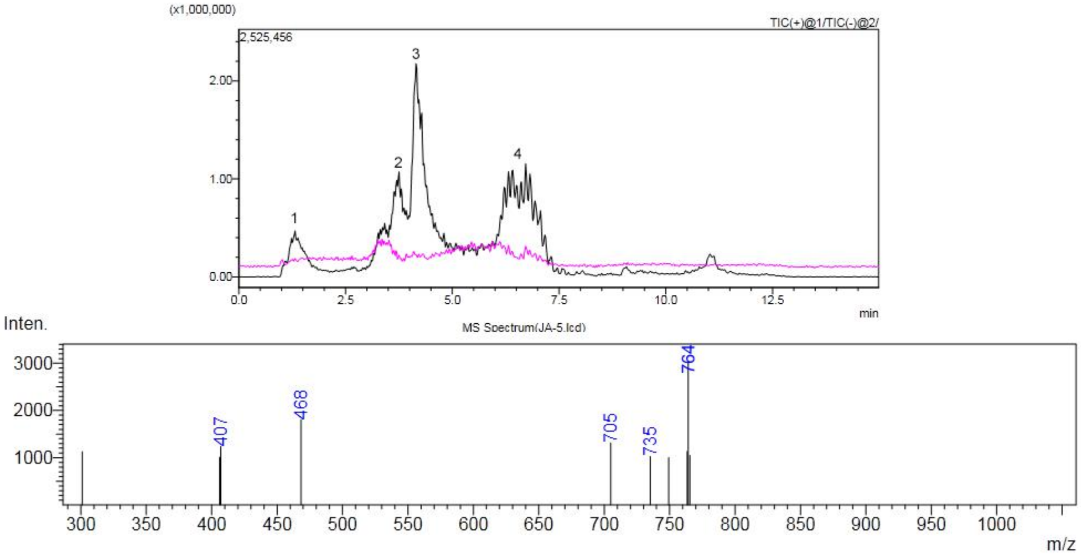

Figure S6. LC-MS analysis of conjugate. Peak 1 represents solvent, peak 2 is unreacted raw material MTX, peak 3 represents the reaction product, and peak 4 represents multimers of starting material.

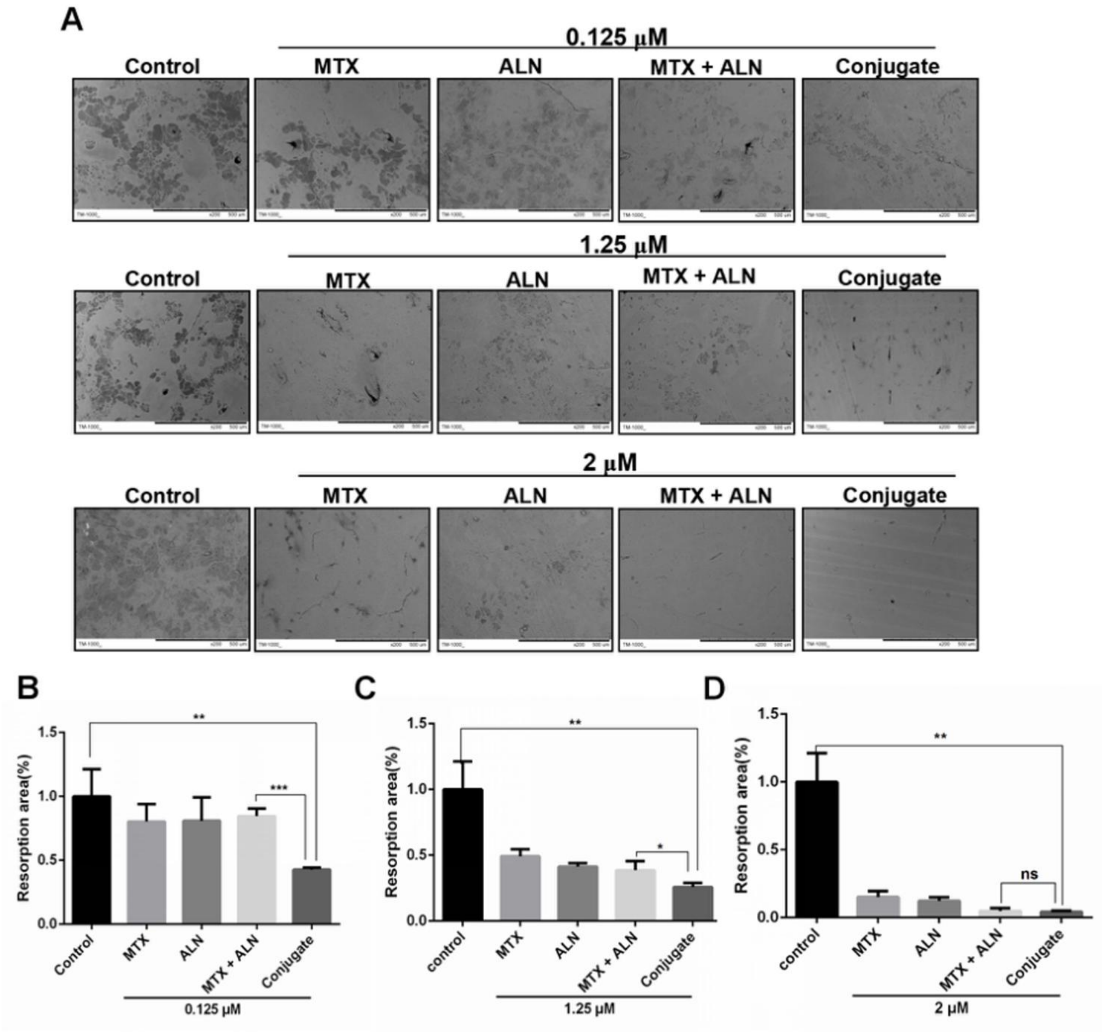

Figure S7. MTX, ALN, MTX with ALN, and MTX-ALN inhibited OC bone resorption in vitro. (A) Scanning electron microscope (SEM) images of bone resorption pits are shown. (B) Resorption pit areas were measured using Image J. ns, not significant. *P<0.05, **P<0.01, ***P<0.005. MTX+ALN, free MTX combined with free ALN.

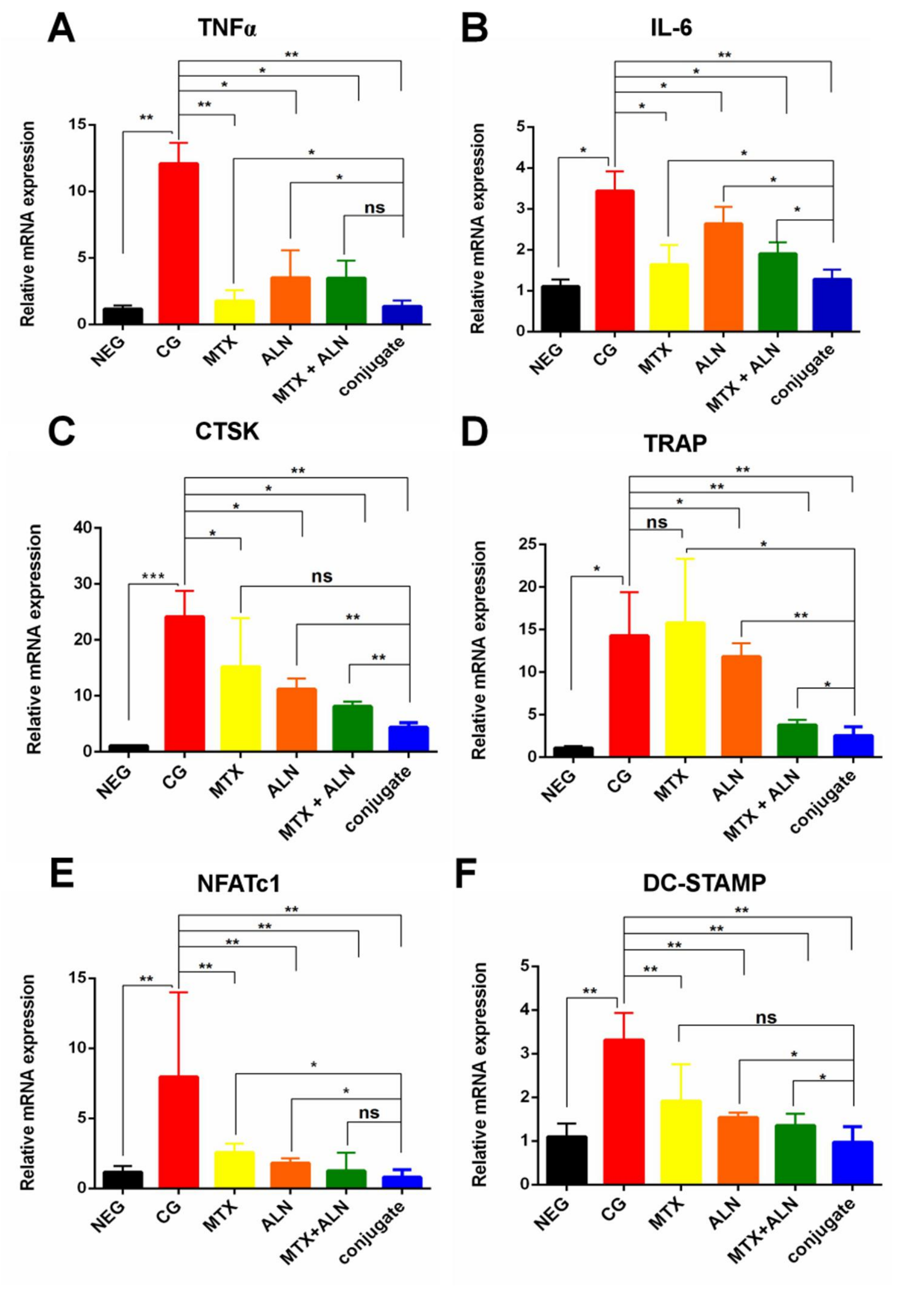

Figure S8. Inflammatory cytokine and OC-related gene expression. (A-F) Total RNA was extracted from the inflamed joints of each rat and reverse transcribed into cDNA. The expression levels of TNFα, IL-6, CTSK, DC-STAMP, NFATc1, and TRAP were examined. *P<0.05, **P<0.01, ***P<0.005. NEG, negative control. CG, control. MTX+ALN, free MTX combined with free ALN.

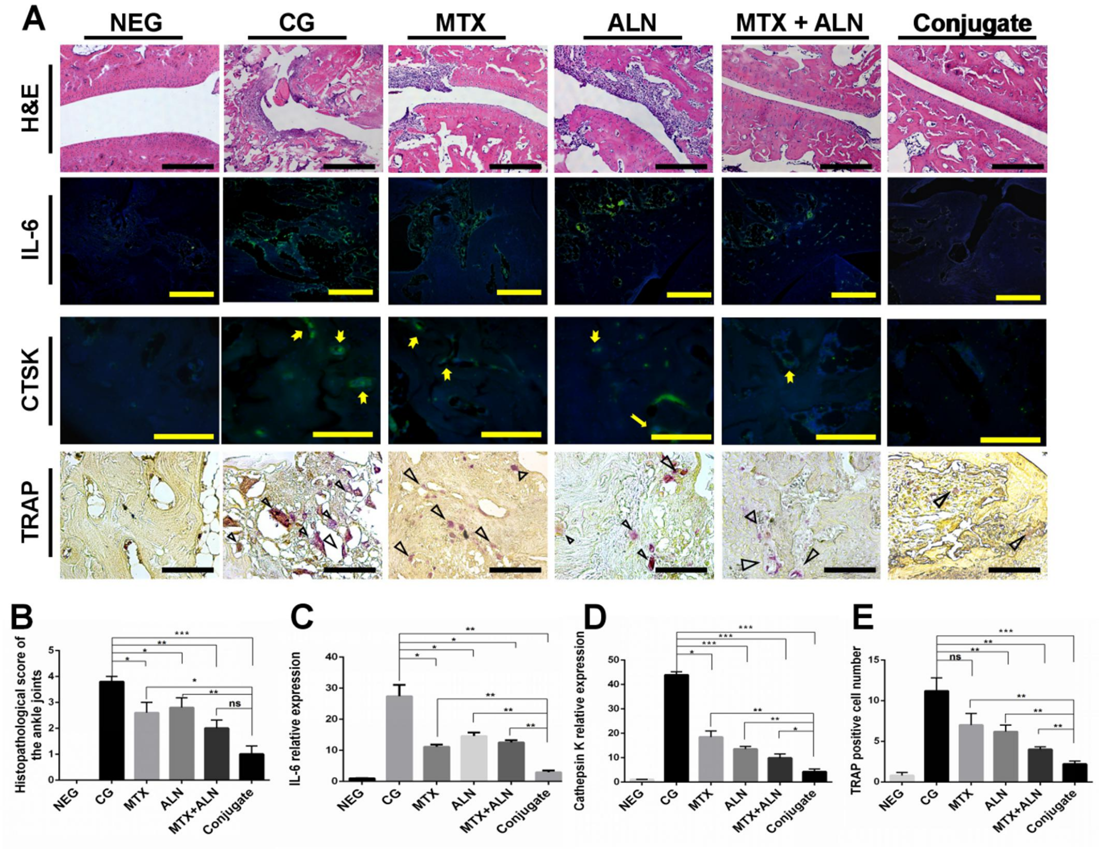

Figure S9. Histological analysis of the CIA model with therapeutic treatment. (A) Joint sections were stained with H&E and immunocytochemistry (yellow arrows, Cathepsin K) was performed to assess the expression of CTSK in OCs of subchondral bones isolated from NEG, CG (CIA models), MTX, ALN, MTX with ALN, and MTX-ALN groups. TRAP staining (black arrows, OCs) showed the extent of bone erosion. (B) Scoring of arthritis on sections stained with H&E. (C) Expression of CTSK and (D) numbers of OCs were measured in sections of subchondral bone. $^{*}P<0.05$, $^{**}P<0.01$, $^{***}P<0.005$. Scale bar = 100 μm. NEG, negative control. CG, control. MTX+ALN, free MTX combined with free ALN.

Supplemental Table 1. Primers used in the real-time PCR

Genes	Upstream (5′-3′)	Downstream (5′-3′)
**GAPDH**	aactcccattcctccacctt	gagggcctctctcttgctct
CTSK	cagcttccccaagatgtgat	tggaggactccagcgtctat
TNF-α	aagctgtcttcaggccaaca	ccgtagggcgattacagtca
IL-6	ctaccccaacttccaatgct	accacagtgaggaatgtcca
NFATc1	tccacccacttctgacttcc	cttcgcccactgatacgag
c-Fos	gttcgtgaaacacaccaggc	ggccttgactcacatgctct
DC-STAMP	gctgtatcggctcatctcct	aaggcagaatcatggacgac
